# Lesser Prairie‐chicken incubation behavior and nest success most influenced by nest vegetation structure

**DOI:** 10.1002/ece3.10509

**Published:** 2023-09-06

**Authors:** Jacquelyn M. Gehrt, Daniel S. Sullins, Bram H. F. Verheijen, David A. Haukos

**Affiliations:** ^1^ Kansas Cooperative Fish and Wildlife Research Unit Kansas State University Manhattan Kansas USA; ^2^ Department of Horticulture and Natural Resources Kansas State University Manhattan Kansas USA; ^3^ Missouri Cooperative Fish and Wildlife Research Unit University of Missouri Columbia Missouri USA; ^4^ U.S. Geological Survey, Kansas Cooperative Fish and Wildlife Research Unit Kansas State University Manhattan Kansas USA

**Keywords:** behavior, incubation, Kansas, Lesser Prairie‐chicken, precipitation, temperature, *Tympanuchus pallidicinctus*, vegetation

## Abstract

Incubation breaks are necessary for any nesting bird but can increase the mortality risk of the nest or attending parent. How intrinsic and extrinsic variables affect nest attentiveness—the proportion of time a female is on nest during incubation— and subsequent survival of the nest remains unclear for uniparental species. We related female nest attentiveness to nest survival and tested the effects of intrinsic and extrinsic variables on nest attentiveness by female Lesser Prairie‐chickens (*Tympanuchus pallidicinctus*) using GPS locations of 87 females at 109 nest sites in 3 study areas in Kansas during 2013–2015. Daily nest survival increased by 39% when nest attentiveness increased from 21% to 98%. Female Lesser Prairie‐chickens were 18% less attentive as body mass increased from 600 to 920 g. Daily precipitation and temperature, controlled for days into the incubation period, had interactive effects on nest attentiveness with nest attentiveness lowest on cool, wet days and increasing as temperature increased, regardless of precipitation (41% attentiveness at 16°C and 79 mm of precipitation to 90% attentiveness at 37°C and 41 mm of precipitation). Nest attentiveness increased by 11% as the quantity of grass at the nest site increased from 5% to 78% when visual obstruction was at 1 and 2 decimeters (dm) and increased 9% as the quantity of grass at the nest site increased from 5% to 83% when visual obstruction was at its maximum (3 dm). Our findings reveal the critical importance of nest attentiveness and incubation behavior, not only in relation to demography, but within the context of changing environmental conditions. As warmer temperatures and extreme precipitation events become more common and change the growth rates of vegetation, species like the Lesser Prairie‐chicken that are ground‐nesting, rely on vegetation cover, and exhibit uniparental care could experience negative demographic consequences.

## Graphical abstract

Over a 3‐year study, we collected movement data of 87 females at 109 nest sites to determine what factors influence incubation behavior and how that influences nest success. We tested both intrinsic and extrinsic factors and found that extrinsic factors, particularly vegetation structure surrounding the nest site influence incubation behavior the most and that incubation behavior does affect nest success.

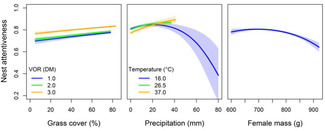

## 1 INTRODUCTION

Incubation of eggs is a critical yet energetically demanding component of parental care among most avian species (Burley & Johnson, 2002; Wesolowski, 2004). In species with uniparental care (~8% of avian species; Cockburn, 2006), the parent must consider the trade‐off between providing for themselves and taking care of the nest. Incubation requires extensive time on the nest to provide thermoregulation (King, 1974) and camouflage (Fontaine & Martin, 2006; Kovarik & Pavel, 2010; Skutch, 1949), for developing eggs; the more time spent on the nest (nest attentiveness), the greater the chances for nest success and offspring survival following hatching (Clutton‐Brock, 1991; Evans & Stutchbury, 2012; Hovick et al., 2014; Nur, 1987; Palmer et al., 2001; Williams, 1996). However, more time invested in nest success means less time available for the parent to prioritize its own survival through finding food and seeking refuge from unfavorable weather and predators (Grisham et al., 2016; Hovick et al., 2014). To adequately balance time between incubation and maintaining body condition, a parent must navigate numerous variables that affect its ability to incubate and be attentive at the nest (Vickery et al., 1999).

Extrinsic variables such as temperature, precipitation, and vegetation structure at the nest can all affect nest attentiveness, particularly for ground‐nesting species (Boal et al., 2014; Conover, 2007; Pitman et al., 2006). If daily temperatures exceed a parent's thermal neutral zone, nest attentiveness could decrease due to an increased need to seek thermal refugia, therefore exposing the nest to extreme temperatures (Boal et al., 2014; Kovarik et al., 2009). Likewise, parents must evaluate the risk of staying on the nest and increasing scent cues that may lead to parent or nest predation during extreme precipitation events with the risk of leaving the nest and exposing it to flooding or egg cooling beyond viability (Conover, 2007; Moynahan et al., 2007; Skagen & Adams, 2012; Webb et al., 2012). Finally, greater vegetation cover and increased structure around a nest can increase protection from aerial and ground predators, especially for species that rely on the cryptic coloration of the parents (Conover et al., 2010; Grisham et al., 2016; Pitman et al., 2006). Increased vegetation cover and structure surrounding the nest may provide enough camouflage to and thermoregulation of the eggs to allow the incubating parent to be less attentive to the nest and prioritize their own survival (Vickery et al., 1999).

The balancing act of parents during incubation may only become more difficult with a changing climate and its environmental effects that may alter the strategies that parents used to raise successful nests. Such environmental effects include increased frequency of extreme weather events, which may create prolonged inhospitable environments for ground‐nesting birds (Fritts et al., 2018). Particularly, unprecedented temperatures and less predictable rainfall may delay vegetation growth and reduce forage resources, which limits quality nest sites that provide available food resources and adequate thermal regulation for nests and incubating parents (Bajgain et al., 2015). Climate change may also affect food resources for avian species as plant phenology may not line up with insect emergence, therefore limiting food availability during the breeding season and negatively affecting the survival of the incubating parent (Kreyling, 2010). Additionally, for a parent exhibiting uniparental care, environmental conditions may cause a shift in priorities to focus on their own survival, further contributing to reduced reproductive output as they risk their survival to maintain viable nesting conditions (DuRant et al., 2019; McKechnie & Wolf, 2010). Ultimately, it is important to acknowledge how these variables differ from species that exhibit biparental incubation to adequately assess how these differing strategies will be affected in the future. Clearly, climate change will influence the extrinsic environment for birds to rear their young, but these changes could also affect bird condition and other intrinsic variables throughout the incubation period.

Intrinsically, bird condition and nesting attempt could affect nest attentiveness (Ardia et al., 2006; Conway & Martin, 2000; Fields et al., 2006; Labocha & Hayes, 2012; Pitman et al., 2006). Female body mass can directly affect the strategy a female uses to thermoregulate eggs. Lighter birds regularly leave the nest to acquire energy through outside food sources (Barzen & Serie, 1990; Drobney, 1982; Krapu, 1981); whereas heavier birds can usually remain in the nest for longer periods by relying on energy reserves during incubation (Cooper, 1978; Raveling, 1979). For birds with multiple nests in a single season, nest attempt itself also affects nest attentiveness as secondary or tertiary nests are often incubated by females in poorer condition (i.e., lighter in mass), typically resulting in increased nest failure (Fields et al., 2006; Gasparini et al., 2006; Lautenbach et al., 2019; Thogmartin & Johnson, 1999).

Most variables affecting nest attentiveness have only been studied in species exhibiting biparental care. For this study, we focused on a short‐lived species with a uniparental mode of care where trade‐offs are a necessary part of the incubation period. Such a species is the Lesser Prairie‐chicken (*Tympanuchus pallidicinctus*), a ground‐nesting prairie grouse. With an average lifespan of ~18 months, Lesser Prairie‐chickens have limited breeding seasons to successfully fledge young and only 1–3 breeding attempts per season (Van Pelt et al., 2013, personal data). Moreover, adult female survival is lowest during the 35‐day nesting and incubation period, increasing mortality risk during this season (Hagen et al., 2007; Jones, 2009; Meyers et al., 2018). Lesser Prairie‐chicken populations exhibit a boom‐bust dynamic, relying on high‐quality nesting habitat, nest success, and brood survival during years with favorable environmental conditions to sustain populations during periods marked by drought conditions, poor nesting conditions, and low recruitment, which would only be exacerbated by climate change (Garton et al., 2016; Hagen et al., 2009; Ross et al., 2018; Sullins et al., 2018; Wisdom & Mills, 1997). Lack of high‐quality habitat was a contributing factor that led this species to be listed under the 1973 Endangered Species Act. Most recently the species was listed as endangered and threatened in their southern and northern Distinct Population Segments, respectively, in 2022 (USFWS, 2022).

Initially, it is important to have a baseline understanding of if and how incubation behavior, particularly nest attentiveness, affects nest success. Such an assessment will improve predictions of how future climate change could affect population demography. We also need to understand which variables drive nest attentiveness to predict if and how these variables may be exacerbated by climate change. To assess how maternal condition, prior nesting attempt, weather, and vegetation structure mediate female incubation behavior and affect nest survival, we fitted female Lesser Prairie‐chickens with GPS transmitters in three study areas in western Kansas during 2013–2015. Our objectives were to (1) evaluate the potential link between nest attentiveness and nest success for Lesser Prairie‐chickens, and (2) assess the relative effects of intrinsic (e.g., body mass, nest attempt, or bird age) and extrinsic variables (e.g., nest vegetation structure, temperature, or precipitation) on nest attentiveness. We hypothesized, based on literature reviews of other bird species, that increased nest attentiveness will indeed lead to increased nest success. We also predicted both extrinsic and intrinsic variables to be important in predicting nest attentiveness as vegetation structure surrounding the nest site is important for camouflage, but energy reserves of the parent are critical in maintaining homeostasis of the nest environment. Overall, these exploratory results will contribute to the understanding of the incubation period ‐ an understudied yet perilous and critical behavior of ground‐nesting birds. In particular, these findings will aid in understanding which intrinsic and extrinsic variables drive incubation behavior in species with uniparental care strategies and give insight into how they could change with a changing climate.

## 2 METHODS

### 2.1 Study area

**FIGURE 1:**
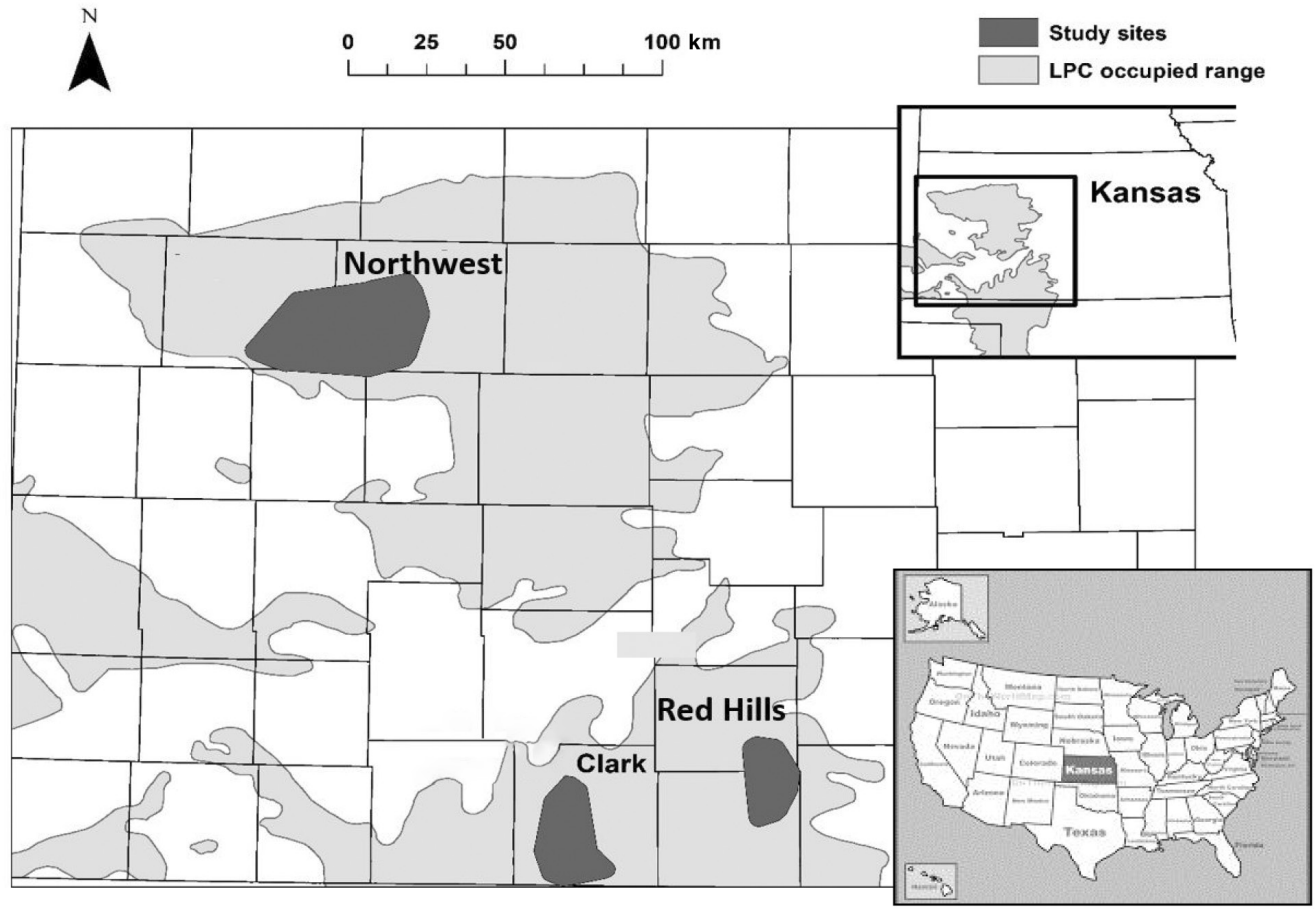
Locations of the three study sites within the Lesser Prairie‐chicken (LPC; *Tympanuchus pallidicinctus*) range in Kansas USA, where nesting female Lesser Prairie‐chickens were tagged with GPS transmitters and monitored during 2013–2015. The Kansas map is depicted with counties, the bottom right corner shows an inset of where Kansas is within the USA and the top right corner has an inset of the LPC range within Kansas.

Our study occurred throughout the northern extent of the current Lesser Prairie‐chicken range in western Kansas from April to July 2013 to 2015 (Figure 1). Study sites encompassed 3 of the 4 ecoregions currently occupied by Lesser Prairie‐chickens including the Mixed‐Grass Prairie and Sand Sagebrush Prairie Ecoregions represented by sites in our Clark study area (37.0733° N, −99.8693° W), the Mixed‐Grass Prairie Ecoregion within the Red Hills (37.3548° N, −99.1204° W) study area, and Short‐Grass Prairie/CRP (Conservation Reserve Program) Mosaic Ecoregion represented by our Northwest study area located within Logan (38.8183° N, −101.621° W) and Gove (38.8539° N, −100.621° W) counties (McDonald et al., 2014). Study sites spanned a precipitation gradient, with the Red Hills study area, the easternmost site, receiving ~690 mm average annual precipitation, and the Northwest study area, the western‐most site, receiving ~370 mm average annual precipitation (PRISM, 2016). These regions were subject to extreme temperature and precipitation events during the study, with at least 4 days per breeding season (May–August) reaching ≥32°C and 3 days per breeding season receiving >50 mm of daily precipitation (Table 1).

**TABLE 1:** Annual precipitation, average yearly temperatures, and range of minimum and maximum temperatures during each year (2013–2015) movements by female Lesser Prairie‐chickens (*Tympanuchus pallidicinctus*) during incubation were monitored in three study sites in Kansas, USA. Weather data obtained from 2020 US Climate Data 2.3.

Study site	Annual precipitation (mm)	Average temperature (°C)	Min. low–max. high (°C)
*Clark*			
2014	600.96	12.77	−8.88 to 34.44
2015	861.06	13.88	−7.77 to 35.00
*Northwest*			
2013	537.97	10.55	−10.55 to 32.22
2014	682.75	10.55	−10.55 to 32.22
2015	448.56	11.66	−8.88 to 33.33
*Red Hills*			
2013	609.09	13.88	−6.11 to 33.33
2014	672.85	13.88	−6.11 to 32.77
2015	856.74	15.55	−5.00 to 34.44

The Clark study site, located in western Clark County, encompassed 712 km^2^ with 77.0% being native grassland, 14.0% cropland, and 5.5% grassland enrolled in CRP (Robinson et al., 2018; Sullins et al., 2018). Dominant land use in Clark included livestock grazing, row‐crop agriculture, and fossil fuel extraction. Average temperature for Clark County during our study (2013–2015) was 13.3°C (US Climate Data, 2022, v3.0). The Red Hills site was located in Comanche and Kiowa counties and encompassed 491 km^2^ of private land comprised of 87.0% native grassland, 8.9% cropland, and 2.2% CRP grassland with livestock grazing, fossil fuel extraction, and limited row‐crop agriculture being the primary land uses (Robinson et al., 2018; Sullins et al., 2018). The Red Hills study site's average temperature during the study was 13.2°C (US Climate Data, 2022, v3.0). The Northwest study site (1714 km^2^) was comprised of 54.0% native grassland, 36.0% cropland, and 7.4% CRP grassland (Robinson et al., 2018; Sullins et al., 2018). This study site was primarily made up of private lands but also included the 69 km^2^ Smoky Valley Ranch, which is owned by The Nature Conservancy. Primary land uses at the Northwest study site included row‐crop agriculture and livestock grazing. Average temperature throughout the study for this study site was 11.5°C (US Climate Data, 2022, v3.0).

### 2.2 Tracking female movements

We trapped Lesser Prairie‐chickens using walk‐in traps (Haukos et al., 1990; Schroeder & Braun, 1991) and drop nets (Silvy et al., 1990) during the lekking season of each year (March–May). We aged birds following capture using plumage characteristics of the outer two primaries (SY [second year] primaries worn and spotted to the tip vs. ASY [after second year] primaries not worn and spotted to within a few cm of the tip) and measured body mass with a spring scale (Ammann, 1944). We fitted females with a rump‐mounted 22‐g Global Positioning System (GPS) satellite transmitter (SAT‐PTT; PTT‐100, Microwave Technology) and tracked them using the GPS/Argos system where locations were recorded every 2 h between 05:00 and 23:00 (10 locations/day). Average body mass was 738 g (range 620–900 g) so the mass of the transmitters was <3.5% of bird mass and did not impede on normal daily activities of female Lesser Prairie‐chickens. We identified the incubation and nesting period based on the female location. If females were at the same location for the majority of the points collected over at least 2 days, we confirmed nest presence and location by flushing the female off nest following Lautenbach et al. (2019).

Subsequently, to distinguish if the bird was on or off nest using GPS locations, we assumed females were on their nest when recorded locations were within 35 m of the nest location while locations outside that range were considered off nest. Our decision of 35 m was based on observed GPS locations from females known to be actively incubating (i.e., females stayed in a single place for a significant proportion of locations received during a 24‐h period, which would be an unanticipated movement pattern for activities such as foraging). Additionally, we confirmed via a histogram that the number of locations recorded within 35 m of the nest was significantly more than those recorded at greater distances away and sharply declined at the 35‐m mark. We believed birds seldom took incubation breaks within 35 m of the nest based on other literature on gallinaceous birds (Bakner et al., 2019; Dudko et al., 2019). We limited our analyses to bird locations that occurred from the initiation of incubation (based on female being in the same location for at least 2 days) to when the nest hatched or failed. We determined the hatch or fail date as the day the female left and never returned to the nest location or the day the female was depredated while still on nest. A nest was deemed successful if at least one egg hatched, which we determined based on the cracking pattern and the presence of an intact membrane within the egg when we went back to the nest site for final fate checks (Lautenbach et al., 2019).

### 2.3 Determining nest attentiveness and distance of and timing of incubation breaks

For each satellite location, we assigned a “1” if the bird was on the nest or a “0” if the bird was off the nest, based on our determined cut‐off distance from nest (35 m). In addition to our primary objectives, we also were interested in basic information about incubation breaks such as the timing of and distances moved from the nest during incubation breaks. We evaluated periods when Lesser Prairie‐chickens were most likely to take incubation breaks and identified incubation breaks as movements with consecutive locations (>2 locations) off nest. We understood that this would be a coarse estimate since our GPS units only collected data every 2 h, but we wanted to test if the bimodal pattern of incubation breaks cited in previous literature was apparent enough to show through despite the lack of fine‐scale frequency of data point collection. We also calculated the distance of incubation breaks as the distance from the recorded nest location to each recorded location of the bird when off the nest (i.e., displacement).

### 2.4 Vegetation sampling

Once a nest hatched, or at the estimated hatch date if a nest had failed, we conducted vegetation surveys at the nest site to evaluate the effect of nest structure on nest attentiveness. At each nest location, we estimated the percent ground cover of grasses, forbs, shrubs, litter, and bare ground using a 60 × 60‐cm modified Daubenmire frame at the center of the point and 4 m in each cardinal direction (Daubenmire, 1959). At each cardinal direction, following Lautenbach et al. (2019), we estimated the height of visual obstruction at 75% obstruction, meaning where the pole was 75% covered by vegetation, to the nearest dm at a height of 1 m using a Robel pole (Robel et al., 1970). We averaged percent ground cover and visual obstruction values recorded among each cardinal direction for one overall value per nest.

### 2.5 Environmental variables

To test the effects of environmental variables on female nest attentiveness, we collected weather data for each day birds were incubating. We obtained daily precipitation (mm) and maximum daily temperature (°C) from the historic records of U.S. climate data (2020 US Climate Data 2.3) and used data from weather stations nearest to our study sites (Red Hills: Coldwater, Kansas; Northwest: Oakley, Kansas; Clark: Ashland, Kansas; Table 1).

### 2.6 Analyses

#### 2.6.1 Relating nest attentiveness patterns to nest success

We used nest survival models to test for the effects of nest attentiveness on daily nest survival using the *RMark* package in R (Laake, 2013). We calculated nest attentiveness as the proportion of locations on nest for each individual bird. Using a hierarchical model selection approach, we tested 9 models, which included single variable, additive, interactive, and intercept‐only variations of nest attentiveness, time as a continuous covariate, time as a discrete covariate, and nest attempt (Table 2). We did not use Bird ID as a random effect because we observed no relationship between female attentiveness on her first versus subsequent nest attempts. For this and all subsequent analyses, we assessed correlations among all proposed variables. If variables were correlated (*r* ≥ .60), we did not use those variables in the same model; however, none of the variables listed above were correlated so our originally hypothesized model sets were not affected. We ranked all models using Akaike's Information Criterion corrected for small sample sizes (AIC_c_; Anderson & Burnham, 2002). If the intercept‐only model was ranked highest, we declared that none of the variables tested within suites were related to nest success. Models within 2 AIC_c_ units of the top‐ranked model were considered to be competing models and potential predictors of nest attentiveness, in which case we used model averaging to determine the most supported predictors.

**TABLE 2:** Ranking of models testing the effects of time (within the breeding season, continuous and discrete), attentiveness (amount of time female is on the nest), and attempt (nesting attempt; first or subsequent) on daily nest survival of female Lesser Prairie‐chicken (*Tympanuchus pallidicinctus*) in Kansas, USA, during 2013–2015.

Model	AIC_c_	ΔAIC_c_	*ω*_*i*_	*K*	Deviance
Time + attentiveness	603.92	0.00	0.35	3	597.91
Attentiveness	604.16	0.23	0.32	2	600.15
Time * attentiveness	605.66	1.74	0.15	4	597.64
Attentiveness + attempt	605.99	2.07	0.13	3	599.98
Attentiveness * attempt	607.64	3.72	0.06	4	599.61
Null	634.96	31.03	0.00	1	632.96
Attempt	636.21	32.29	0.00	2	632.21
Time (continuous)	636.79	32.87	0.00	2	623.78
Time (discrete)	702.32	98.39	0.00	75	545.84

*Note*: The additive model of time and attentiveness was most supported, but time was considered spurious with little influence on daily nest survival. AIC_c_ = Akaike's Information Criterion adjusted for sample size, ΔAIC_c_ = difference in AIC_c_ relative to smallest AIC_c_ value, *ω*_*i*_ = AIC_c_ weight, *K* = no. of parameters, Deviance = model fit.

#### 2.6.2 Intrinsic and extrinsic variables of nest attentiveness

We used logistic regression models to test for the effects of intrinsic and extrinsic variables on female nest attentiveness. We tested 4 separate model suites (vegetation, condition of female, environmental, and descriptive), each of which except descriptive included single variable, quadratic, additive, interactive (limited to two‐way interactions), and intercept‐only models (Table S1). For several variables where it made ecological sense (i.e., we believed the response of the Lesser Prairie‐chickens would not be a strict positive linear relationship, for instance, as temperature or precipitation increased beyond a certain threshold or visual obstruction became too dense), we tested the relevant quadratic term. The first model suite included the condition of the females (age [SY vs. ASY] and body mass) for a total of 7 models. We hypothesized that with age would come more experience and perhaps more dedication to nest attentiveness with increasing age and decreasing nesting chances. Additionally, we thought body mass would be a proxy for female condition going into the incubation stage. The more energy reserves (measured as more mass) a bird has, the less time she would need to search for food and instead be incubating the nest. The second model suite tested environmental variables including maximum daily temperature and daily precipitation for a total of 13 models. Although the correlation between temperature and time since incubation start was negligible (0.10), we tested one a posteriori model accounting for time since initiation to potentially account for temperature due to the temporal variation of this variable as the nesting season progresses. We hypothesized temperature and precipitation to be important predictors of nest attentiveness because as either one becomes more extreme, an incubating female may shift her priorities to care for herself more. For example, as temperature becomes extreme or precipitation levels climb higher, females may abandon the nest to seek thermal refugia or refuge from the precipitation, respectively, and prioritize their own survival. The third model suite included vegetation variables including 75% visual obstruction, percent ground cover of grasses, percent ground cover of forbs, and percent ground cover of shrubs at the nest site for a total of 15 models. We used these variables because in previous research it has been found that visual obstruction affects nest survival as it provides concealment from predators and the elements. Additionally, green vegetation absorbs more energy from the sun than bare ground, so we combined visual obstruction with other vegetation cover at the nest site to determine if one or both types of vegetation categories would be the best determinant of nest attentiveness. For both the second and third model suites, we truncated values at the minimum and maximum values of a minimum convex polygon fitted to plotted values to ensure trends depicted true scenarios experienced in the field during the study. The last model only included the descriptive variables of nest attempt and time into the incubation period against the null model. We then combined the top models from each suite (4 models) into one final suite to determine the most parsimonious model. We ranked all models based on parsimony using AIC_c_ and used model selection criteria described above related to nest success models (Anderson & Burnham, 2002).

## 3 RESULTS

**FIGURE 2:**
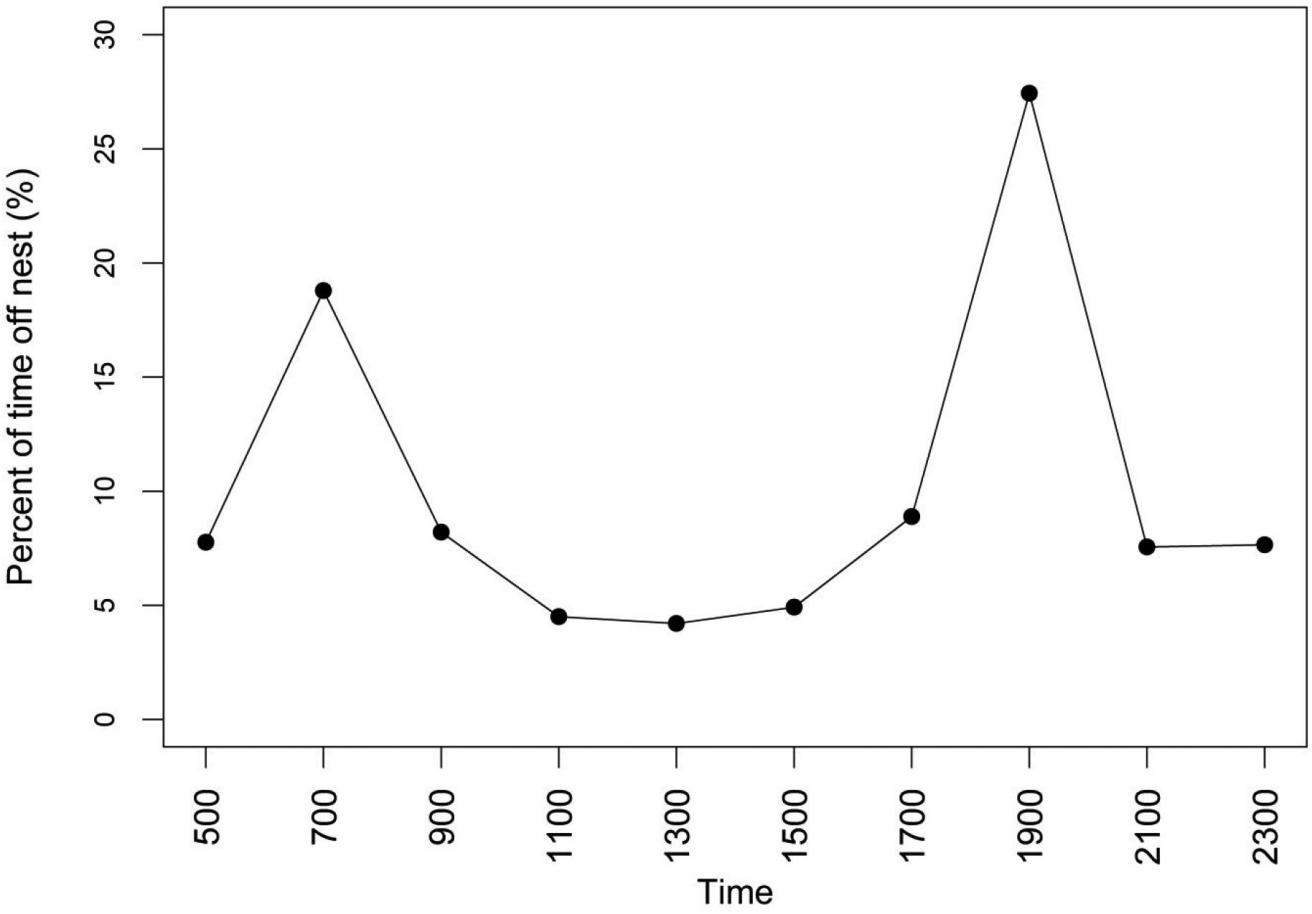
Percent of time female Lesser Prairie‐chickens (*Tympanuchus pallidicinctus*) were off nest (*n* = 3689 off‐nest locations) from 0500 to 2300 at three study sites in Kansas, USA, during 2013–2015.

**FIGURE 3:**
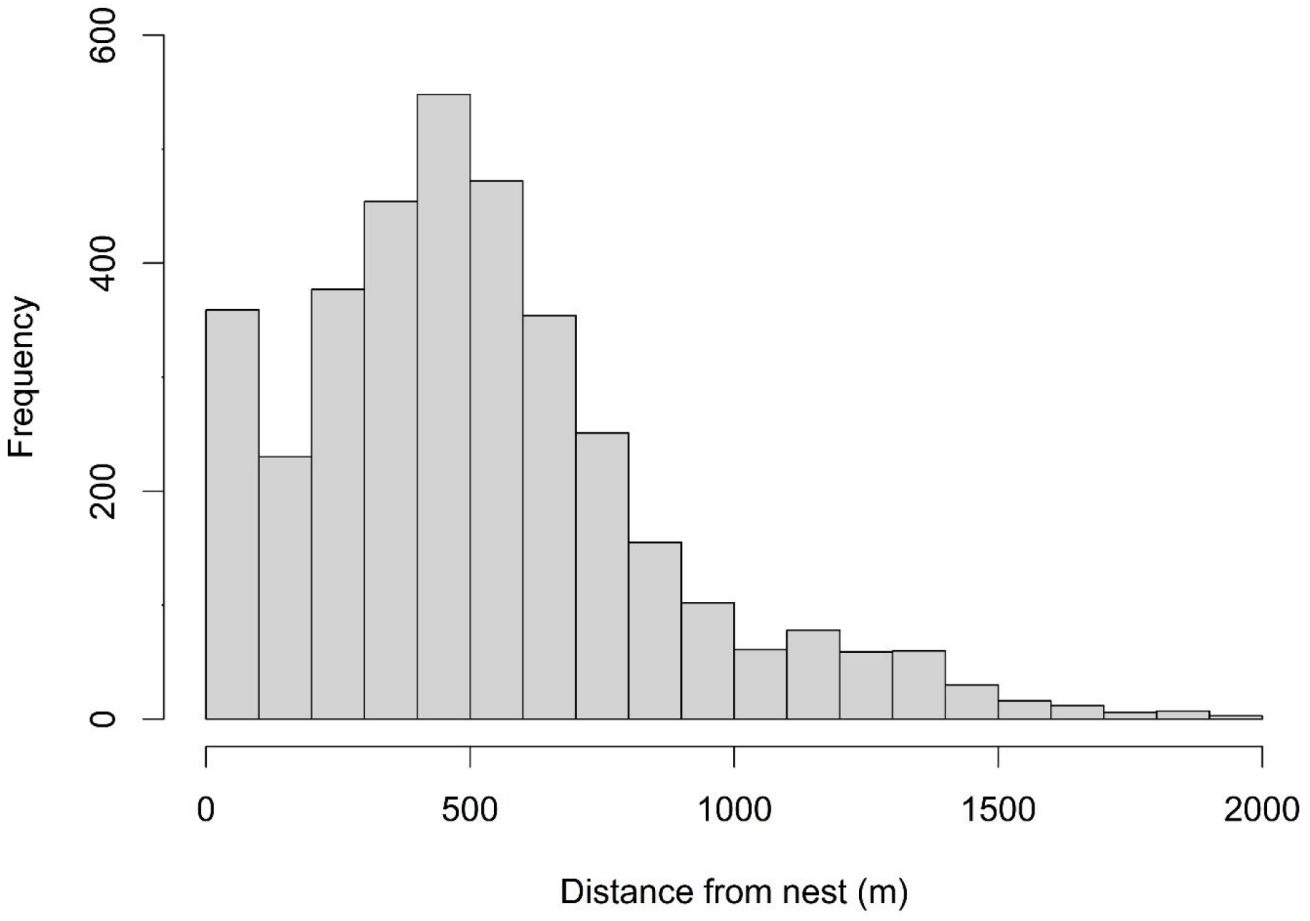
Distance traveled from the nest by female Lesser Prairie‐chickens (*Tympanuchus pallidicinctus*) during incubation breaks (*n* = 3689 off‐nest locations) at three study sites in Kansas, USA, during 2013–2015.

We tracked 87 female Lesser Prairie‐chickens throughout the study, resulting in the monitoring of 109 nests (85 first and 24 subsequent attempts), with 69 nests from SY birds and 40 from ASY birds (Table 3). Out of 109 nests, 34 were successful and 75 failed. During the incubation period, we recorded 17,574 GPS locations with 3689 being off‐nest locations. Mean attentiveness for first nest attempts was 74.10% ± 1.68% (SE; range: 26.92%–98.51%), while for subsequent nest attempts, it was 77.64% ± 2.17% (SE; range: 52.69%–90.63%). We observed a large percentage of incubation breaks at dawn and dusk; nearly 28% of all recorded breaks were taken at 19:00 and 20% at 07:00 (Figure 2). Identified incubation breaks were often 400–600 m from the nest location ($\bar{x}$ = 545 ± 7 m; Figure 3).

**TABLE 3:** Counts of nests by attempt (first, second, or third), age (SY = second year, ASY = after second year), study site, and year for female Lesser Prairie‐chickens (*Tympanuchus pallidicinctus*) monitored in Kansas, USA, during 2013–2015.

Study site	ASY			SY		
	First	Second	Third	First	Second	Third
*Clark*						
2014	2	1	0	5	1	0
2015	2	0	0	6	0	0
*Northwest*						
2013	4	1	0	10	0	0
2014	10	2	0	7	3	0
2015	2	1	0	9	2	0
*Red Hills*						
2013	2	0	0	7	1	0
2014	6	4	1	8	4	1
2015	2	0	0	3	2	0
Total	30	9	1	55	13	1

**FIGURE 4:**
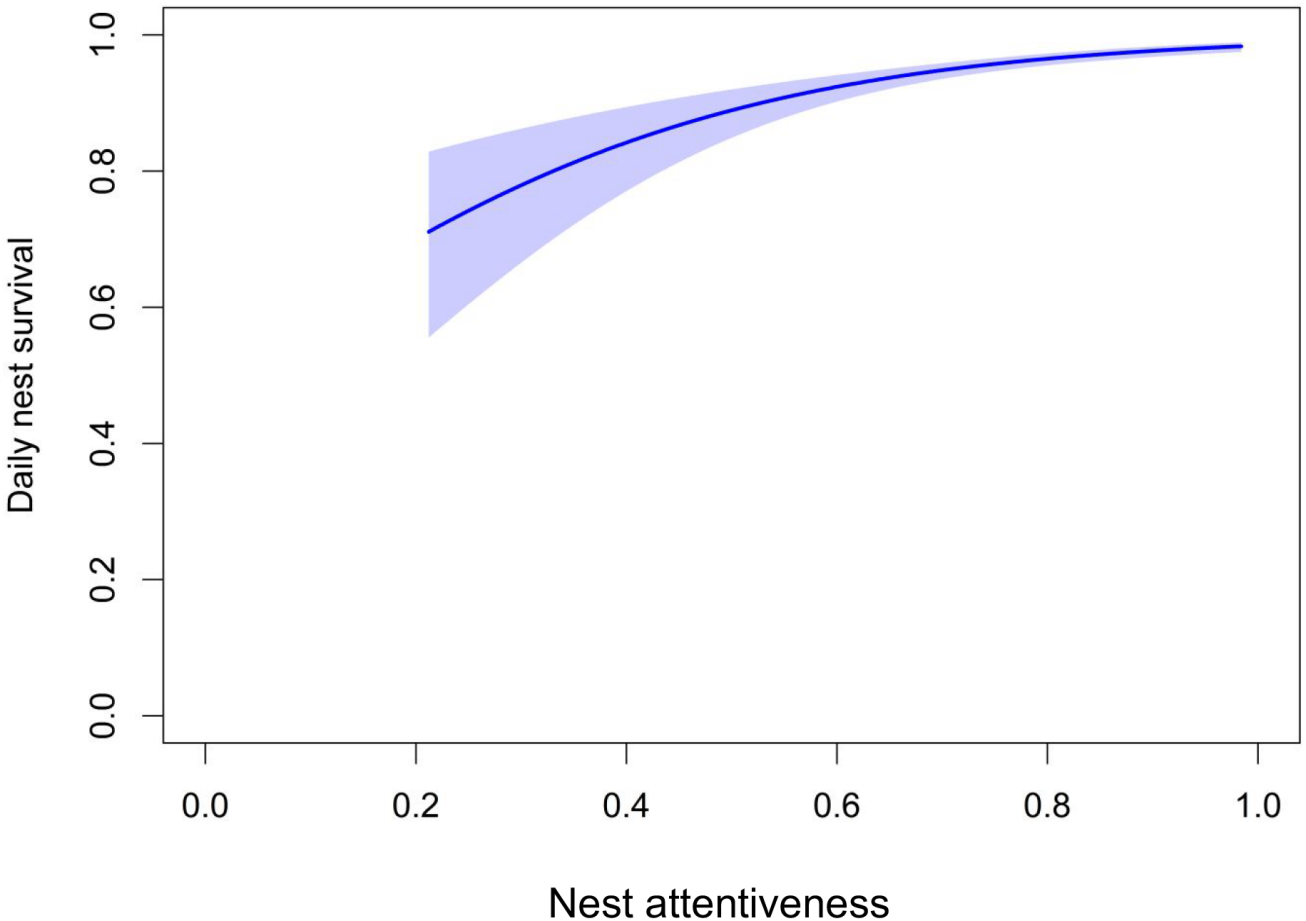
Daily nest survival of female Lesser Prairie‐chickens (*Tympanuchus pallidicinctus*) as a function of their nest attentiveness, depicted as a proportion of the on‐ versus off‐nest locations per bird) throughout three study sites in Kansas, USA during 2013–2015, shown with 95% confidence intervals.

Nest attentiveness had a positive effect on daily nest survival (*β*_attentiveness_ = 4.11 ± 0.64; Table 2, Figure 4), with daily nest survival increasing by 39% when nest attentiveness increased from its minimum (21%) to its maximum (98%) value. When examining variables affecting nest attentiveness, we observed that extrinsic variables related to vegetation surrounding nests were most influential to female presence at nest, specifically visual obstruction and percent ground cover of grass at the nest site (*ω*_*i*_ = 0.53; Table 4). Within each of the other categories, female attentiveness was best predicted by the environmental variables of daily precipitation and temperature accounting for time into the incubation period (*ω*_*i*_ = 1.00), condition variable of female body mass at time of capture (ω_i_ = 0.33), and time into the incubation period among descriptive characteristics (*ω*_*i*_ = 0.84; Table S1).

**TABLE 4:** Ranking of all top‐ranking models from vegetation (visual obstruction reading [VOR], grass cover), condition (body mass), environmental (precipitation and temperature accounting for time), and descriptive (days into incubation period) model suites to determine the best predictor of female Lesser Prairie‐chicken (*Tympanuchus pallidicinctus*) nest attentiveness in Kansas, USA, during 2013–2015 indicated that vegetation characteristics surrounding the nest had the great influence on percent time spent on nests during incubation.

Model	AIC_c_	ΔAIC_c_	*ω*_*i*_	*K*	Deviance
VOR^2^ + percent ground cover grass	15,973.16	0.00	1.00	4	−7982.58
Precipitation^2^ ×Temerature^2^ (time)	17,248.96	1275.80	0.00	7	−8617.48
Time	17,293.13	1319.97	0.00	2	−8644.56
Mass^2^	17,956.12	1982.96	0.00	3	−8975.06
Null	18,024.50	2051.34	0.00	1	−9011.25

*Note*: AIC_c_ = Akaike's Information Criterion adjusted for sample size, ΔAIC_c_ = difference in AIC_c_ relative to smallest AIC_c_ value, *ω*_*i*_ = AIC_c_ weight, *K* = no. of parameters, Deviance = model fit.

Abbreviation: VOR, visual obstruction reading.

**FIGURE 5:**
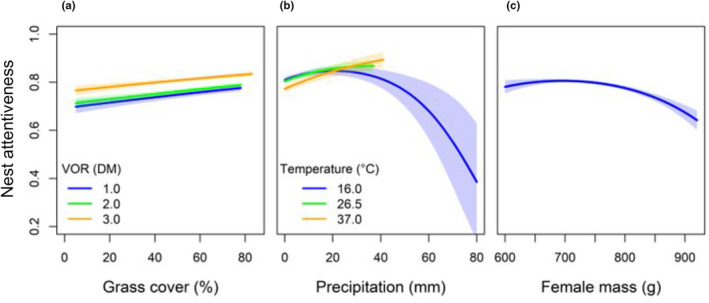
Nest attentiveness of female Lesser Prairie‐chickens (*Tympanuchus pallidicinctus*) as a function of the most supported models within the vegetation (a), environmental (b), and condition (c) model suites. (a) Nest attentiveness increased as grass cover around the nest site increased, increasing slightly with taller VOR. (b) Nest attentiveness of female Lesser Prairie‐chickens increased regardless of precipitation values when temperatures were warm, but when temperatures were at minimum values, attentiveness dropped significantly as precipitation increased beyond a certain threshold. (c) Nest attentiveness of female Lesser Prairie‐chickens decreased as female mass at time of capture increased. All trends are shown with 95% confidence intervals and depict nest attentiveness trends of females throughout three study sites in Kansas, USA, during 2013–2015. Graphs a and b are shown with minimum, mean, and maximum values of the range of visual obstruction and temperature, respectively.

With respect to vegetation variables, female attentiveness increased as visual obstruction and grass cover surrounding the nest increased, with grass cover having more influence when visual obstruction was ≤2 dm (*β*_VOR_^2^ = 0.1021 ± 0.0249, *β*_grass_ = 0.0055 ± 0.0009). Female attentiveness increased by 11% as the amount of grass at the nest site increased from 5 to 78% when visual obstruction was at its minimum (1.0 dm) and mean (2.0 dm) value, but only increased by 9% when visual obstruction was at the maximum value (3.0 dm; Figure 5a). Among the most supported environmental variables, females were less attentive at the nest as precipitation increased, but only during low temperatures (*β*_precip_ = 0.0356 ± 0.0050, *β*_precip_^2^ = −0.0011 ± 0.0002, *β*_temp_ = 0.0325 ± 0.0161, *β*_temp_^2^ = −0.0007 ± 0.0003, *β*_precip_^2^_× temp_^2^ = 0.0000 ± 0.0000). When daily maximum temperatures were at the minimum value (16°C), nest attentiveness decreased by 55% as daily precipitation amounts increased from 0 to 80 mm whereas when daily maximum temperatures were at the maximum value (37°C), nest attentiveness increased by 19% when daily precipitation increased from 0 to 41 mm (Figure 5b). The most supported model in the condition suite without spurious variables was a single‐variable model of body mass in the quadratic form. Unexpectedly, this model depicted nest attentiveness to decrease as body mass increased, with females 22% less attentive as mass increased from 700 to 920 g (*β*_mass_ = 0.0225 ± 0.0049, *β*_mass_^2^ = 0.0000 ± 0.0000; Figure 5c).

## 4 DISCUSSION

Our findings add to the limited knowledge of incubation behavior for avian species, particularly in uniparental ground‐nesting birds. We found a number of intrinsic and extrinsic variables can affect incubation behavior, including vegetation, weather, and female condition. These results highlight the complex interaction among environmental conditions, nest site selection, and female behavior that plays a critical role in nest success for an imperiled species.

We documented Lesser Prairie‐chickens were most likely to take incubation breaks at dawn and dusk and move on average 400–600 m from the nest during breaks. Observed timing of incubation breaks was consistent with previous studies focused on grouse species, which highlighted bimodal trends in incubation break timing centered around sunrise and sunset (Coates & Delehanty, 2008; Summers et al., 2009; Wiebe & Martin, 1998; Winder et al., 2016). Most studies showed birds to take breaks anywhere from 30 min to several hours after sunrise and 30–90 min before sunset when it was still light enough to forage, but where birds could return to nests under low‐light conditions to minimize attraction to the nest location (Martin, 2002). Timing of incubation breaks has been proposed to be related to a) the metabolic need of the incubating parent (Wiebe & Martin, 1998; Winder et al., 2016), and b) avoidance of nest exposure to high temperatures, which threatens embryo development more so than low temperatures (Grisham et al., 2013, 2016; Webb, 1987). Because birds cease foraging during low and no‐light hours, morning and evening incubation breaks may align with the depletion of energy reserves after fasting all night and all day, respectively. An additional reason may be due to the temperature trends during the nesting season, when the hottest temperatures occur during the middle of the day and embryo viability is most at risk (Grisham et al., 2016).

Our estimates of mean nest attentiveness and distance traveled during incubation breaks differed from other grouse studies. Attentiveness levels were lower in our research than in other prairie grouse studies (74.10%–77.64% nest attentiveness vs. 96% and 95%; Coates & Delehanty, 2008; Winder et al., 2016). It should be noted, however, that these two studies used recording devices to obtain more precise data than our GPS transmitters, which only recorded one data point every 2 h, so attentiveness may have been greater than we were able to determine with our recorded intervals. Several studies have sought to examine the distance birds traveled during incubation breaks and highlight the connection between distance traveled during incubation breaks and the accessibility and availability of resources on the landscape (Conley et al., 2015; Dudko et al., 2019). Greater Sage‐grouse (*Centrocercus urophasianus*) hens in central Nevada had average movements of 242 m from the nest during incubation breaks (Dudko et al., 2019). Conley et al. (2015) found that Rio Grande turkeys (*Meleagris gallopavo*) moved on average less than 100 m from their nests on incubation breaks. We discovered Lesser Prairie‐chickens moved further, typically 400–600 m from the nest during the majority of incubation breaks. This aspect of the incubation period needs additional study to evaluate resource use during breaks. Implications of these studies could broaden the definition of what constitutes the spatial scale of nesting habitat to ensure incubating hens can obtain necessary resources during this stage.

We found that variation in nest attentiveness by female Lesser Prairie‐chickens does indeed affect nest fate, similar to findings from Bakner et al. (2019) and Lohr et al. (2020) for Wild Turkey (*Meleagris gallopavo*). Although nest attentiveness is significantly affected by both intrinsic and extrinsic variables, vegetation variables at the nest site had the greatest influence on nest attentiveness by female Lesser Prairie‐chickens and may be key to balancing the competing priorities of females during incubation. Lesser Prairie‐chickens generally select for and have greater nest success at nest sites with high visual obstruction (Grisham et al., 2016; Hagen et al., 2004; Lautenbach et al., 2019), but the amount of horizontal grass cover at the nest site can partially offset negative effects of short visual obstruction on nest success. Grass absorbs more solar energy than bare ground and can not only provide a better environment for growing embryos, but also provide thermal refuge for the incubating parent via evapotranspiration (McIlroy & Slatyer, 1961). Managing for amount and height of grass for Lesser Prairie‐chicken nesting habitat could therefore provide incubating females with an environment that can maximize their time on the nest while also maintaining their body condition (Boal et al., 2014; Grisham et al., 2016).

Vegetation management may also assist females in navigating environmental variables, such as temperature and precipitation, particularly when temperatures are high during the incubation period. We found an interacting effect between temperature and precipitation on incubation patterns with nest attentiveness being greater during warmer temperatures regardless of precipitation events. One explanation for this pattern may be that higher temperatures were often experienced later in incubation or during renesting (e.g., first nest attempt avg. ± SE = 25.6 ± 0.1°C versus subsequent attempts = 31.2 ± 0.1°C) when females may be more likely to prioritize hatch success over their own survival, even if a rain event were to occur (Bakner et al., 2019; Becker & Zhang, 2011; Brunton, 1988; Wendeln et al., 1999). We assessed the relationship between temperature and days into the incubation period and found a slight positive relationship (*r* = .10) between these two variables. We ended up controlling for this variable in our ‘*temperature × precipitation*’ model to ensure temperature was not a spurious effect of time. Grisham et al. (2016) found similar trends of greater investment by the female during hotter temperatures; they found for every 30 min a nest was exposed to temperatures above 34°C, the probability of nest survival decreased by 10%. Alternatively, the effects of precipitation on nesting females may be moderated during warm weather due to their ability to thermoregulate themselves and the nest more effectively. Our modeled relationships were based on linear relationships to weather and future research might benefit from testing extreme weather events separately as these events may influence avian nest success but remain unclear (Conover, 2007; Fogarty et al., 2017; Skagen & Adams, 2012).

Among intrinsic variables, we unexpectedly found Lesser Prairie‐chicken nest attentiveness to decrease with increasing body mass at time of capture (March–April). In many other studies, and what we predicted, increased body mass of the incubating parent often allows for greater nest attentiveness due to increased energy reserves, therefore increased nest success and lower rates of nest abandonment (Aldrich & Raveling, 1983; Dearborn, 2001; Wendeln et al., 1999; Yorio & Boersma, 1994). Our hypothesized relationship was not confirmed, so an alternative hypothesis could be that increasing body mass allows females enough nutrient or energy reserves to leave the nest and explore potential brood areas, feed, or seek refuge from unfavorable environmental conditions although no other study has yet to test this aspect of incubation.

Our paper joins many existing studies that emphasize how various life history stages of bird species will be affected in the near future due to the effects of climate change. This is especially true with Lesser Prairie‐chickens as we found the most supported variables determining nest attentiveness to be extrinsic (vegetation and precipitation/temperature) versus intrinsic (mass at time of capture). The effects these variables have on nest attentiveness could be accentuated in the future due to rising maximum temperatures and more sporadic and extreme precipitation events (Ojima et al., 2021; Skagen & Adams, 2012). As these events become more common, they could lead to damaging results for Lesser Prairie‐chicken populations (widespread nest abandonment/destruction and decreased seasonal fecundity). Additionally, predicted changes in climate will likely alter plant phenology and growth, with lower growth rates and delayed emergence during drought conditions (Grisham et al., 2013; Kreyling, 2010). These slower growth rates may also lead to delayed onset of incubation, which has been found to decrease nest survival (Fields et al., 2006; Pitman et al., 2006). Our findings indicate that the effects of climate change currently being experienced as well as those predicted to occur in the future necessitate prudence and foresight of grassland managers to curb the most deleterious effects on Lesser Prairie‐chickens.

## AUTHOR CONTRIBUTIONS

**Jacquelyn M. Gehrt:** Conceptualization (equal); formal analysis (equal); investigation (lead); writing – original draft (lead). **Daniel S. Sullins:** Conceptualization (equal); investigation (lead); writing – review and editing (equal). **Bram H. F. Verheijen:** Formal analysis (equal); writing – review and editing (equal). **David A. Haukos:** Resources (lead); supervision (lead); writing – review and editing (equal).

## FUNDING INFORMATION

We thank the people and agencies that provided funding for this project including C. Hagen and J. Pitman, the Federal Aid in Wildlife Restoration Grant W‐73‐R; US Geological Survey; US Dept. of Agriculture (USDA), Natural Resources Conservation Service, Lesser Prairie‐Chicken Initiative; Kansas Wildlife and Parks (Federal Assistance Grant KS W‐73‐R‐3); USDA Farm Services CRP Monitoring, Assessment, and Evaluation (12‐IA‐MRE CRP TA#7, KSCFWRU RWO 62). No funders had any input in the content of this manuscript, nor did they require their approval before submission.

## CONFLICT OF INTEREST STATEMENT

There are no conflicts of interest that we know of for this manuscript.

## Supporting information


Table S1

## Data Availability

Available from the corresponding author upon reasonable request due to use of sensitive location data of a threatened species.
