# Supplementary material for: Lesser Prairie‐chicken incubation behavior and nest success most influenced by nest vegetation structure
Source: Ecol Evol. 2023 Sep 6;13(9):e10509. doi: 10.1002/ece3.10509 (PMC10483095; doi:10.1002/ece3.10509)
Supplement: Supplementary file 1 — Table S1 [file ECE3-13-e10509-s001.docx]

# **SUPPLEMENTAL TABLES**

**Table S1.** Complete model sets used to evaluate vegetation, condition, environmental, and descriptive variables as predictors of nest attentiveness by female Lesser Prairie-chickens (*Tympanuchus pallidicinctus*) in Kansas, USA, during 2013–2015.

|  | Model | AICc^1^ | ΔAICc | | ω_i_ | K | Deviance |
| --- | --- | --- | --- | --- | --- | --- | --- |
| ***Vegetation Variables*** | |  |  |  | |  |  |
|  | VOR^2^ + percent ground cover grass | 15973.16 | 0.00 | | 0.51 | 4 | -7982.58 |
|  | VOR^2^ × percent ground cover grass | 15973.61 | 0.44 | | 0.41 | 5 | -7981.80 |
|  | VOR × percent ground cover grass | 15978.10 | 4.94 | | 0.04 | 4 | -7985.05 |
|  | VOR^2^ + percent ground cover forb | 15979.46 | 6.29 | | 0.02 | 4 | -7985.73 |
|  | VOR^2^ x percent ground cover forb | 15980.61 | 7.45 | | 0.01 | 5 | -7985.30 |
|  | VOR + percent ground cover grass | 15988.50 | 15.34 | | 0.00 | 3 | -7991.25 |
|  | VOR^2^ * percent ground cover shrub | 15999.88 | 26.72 | | 0.00 | 5 | -7994.90 |
|  | VOR * percent ground cover forb | 16001.34 | 28.17 | | 0.00 | 4 | -7996.67 |
|  | VOR^2^ + percent ground cover shrub | 16001.82 | 28.66 | | 0.00 | 4 | -7996.91 |
|  | VOR + percent ground cover forb | 16003.35 | 30.18 | | 0.00 | 3 | -7998.67 |
|  | VOR^2^ | 16003.94 | 30.77 | | 0.00 | 3 | -7998.97 |
|  | VOR * percent ground cover shrub | 16016.65 | 43.48 | | 0.00 | 4 | -8004.32 |
|  | VOR + percent ground cover shrub | 16021.06 | 47.89 | | 0.00 | 3 | -8007.53 |
|  | VOR | 16023.51 | 50.35 | | 0.00 | 2 | -8009.75 |
|  | Null | 16072.88 | 99.72 | | 0.00 | 1 | -8035.44 |
| ***Condition Variables*** |  |  |  | |  |  |  |
|  | Age × mass^2^ | 17954.70 | 0.00 | | 0.67 | 5 | -8972.35 |
|  | Mass^2^ | 17956.12 | 1.43 | | 0.33 | 3 | -8975.06 |
|  | Mass × age | 17964.46 | 9.77 | | 0.00 | 4 | -8978.23 |
|  | Mass + age | 17977.78 | 23.08 | | 0.00 | 3 | -8985.88 |
|  | Mass | 17980.34 | 25.64 | | 0.00 | 2 | -8988.17 |
|  | Null | 18024.50 | 69.81 | | 0.00 | 1 | -9011.25 |
|  | Age | 18025.84 | 71.15 | | 0.00 | 2 | -9010.92 |
| ***Environmental Variables*** |  |  |  | |  |  |  |
|  | Precip^2^ × temp^2^ + time | 17248.96 | 0.00 | | 1.00 | 7 | -8617.48 |
|  | Precip^2^ × temp^2^ | 17959.10 | 710.14 | | 0.00 | 6 | -8973.54 |
|  | Precip^2^ × temp | 17961.74 | 712.78 | | 0.00 | 5 | -8975.87 |
|  | Temp × precip^2^ | 17974.34 | 725.38 | | 0.00 | 5 | -8982.17 |
|  | Precip^2^ | 17979.42 | 730.46 | | 0.00 | 3 | -8986.71 |
|  | Precip^2^ + temp | 17981.42 | 732.46 | | 0.00 | 4 | -8986.71 |
|  | Temp^2^ × precip | 17988.35 | 739.39 | | 0.00 | 5 | -8989.17 |
|  | Temp × precip | 17991.07 | 742.10 | | 0.00 | 4 | -8991.53 |
|  | Temp^2^ + precip | 17992.65 | 743.69 | | 0.00 | 4 | -8992.32 |
|  | Precip | 17998.26 | 749.30 | | 0.00 | 2 | -8997.13 |
|  | Temp × Precip | 18000.09 | 751.13 | | 0.00 | 3 | -8997.05 |
|  | Temp | 18018.40 | 769.44 | | 0.00 | 3 | -9006.20 |
|  | Null | 18024.50 | 775.54 | | 0.00 | 1 | -9011.25 |
|  | Temp | 18025.51 | 776.55 | | 0.00 | 2 | -9010.76 |
| ***Descriptive Variables*** |  |  |  | |  |  |  |
|  | Time | 17293.13 | 0.00 | | 0.84 | 2 | -8644.56 |
|  | Time*Attempt | 17296.51 | 3.37 | | 0.16 | 4 | -8644.25 |
|  | Attempt | 18022.87 | 729.74 | | 0.00 | 2 | -9009.42 |
|  | Null | 18024.50 | 731.37 | | 0.00 | 1 | -9011.25 |
|  |  |  |  | |  |  |  |
|  |  |  |  | |  |  |  |

^1^ AIC_c_ = Akaike’s Information Criterion adjusted for sample size, ΔAICc = difference in AICc relative to smallest AICc value, ωi = AICc weight, K = no. of parameters, Deviance = model fit, VOR= Visual Obstruction Reading, Precip = Precipitation, temp = Temperature
